# Antimycobacterial Precatorin A Flavonoid Displays Antibiofilm Activity against *Mycobacterium bovis* BCG

**DOI:** 10.1021/acsomega.3c05703

**Published:** 2023-10-20

**Authors:** Esmeralda Ivonne Niño-Padilla, Clara Espitia, Carlos Velazquez, Efrain Alday, Erika Silva-Campa, Alexel Burgara-Estrella, José Antonio Enciso-Moreno, Olivia Valenzuela, Humberto Astiazarán-García, Adriana Garibay-Escobar

**Affiliations:** † Departamento de Ciencias Químico Biológicas, Universidad de Sonora, Rosales y Luis Encinas s/n, Hermosillo 83000, Sonora, México; ‡ Departamento de Inmunología, Instituto de Investigaciones Biomédicas, Universidad Nacional Autónoma de México, Ciudad Universitaria, Coyoacán 04510, Ciudad de México, México; § Departamento de Investigación en Física, Universidad de Sonora, Rosales y Luis Encinas s/n, Hermosillo 83000, Sonora, México; ∥ Facultad de Química, Universidad Autónoma de Querétaro, Centro Universitario s/n, Cerro de las Campanas, Santiago de Querétaro 76010, Querétaro, México

## Abstract

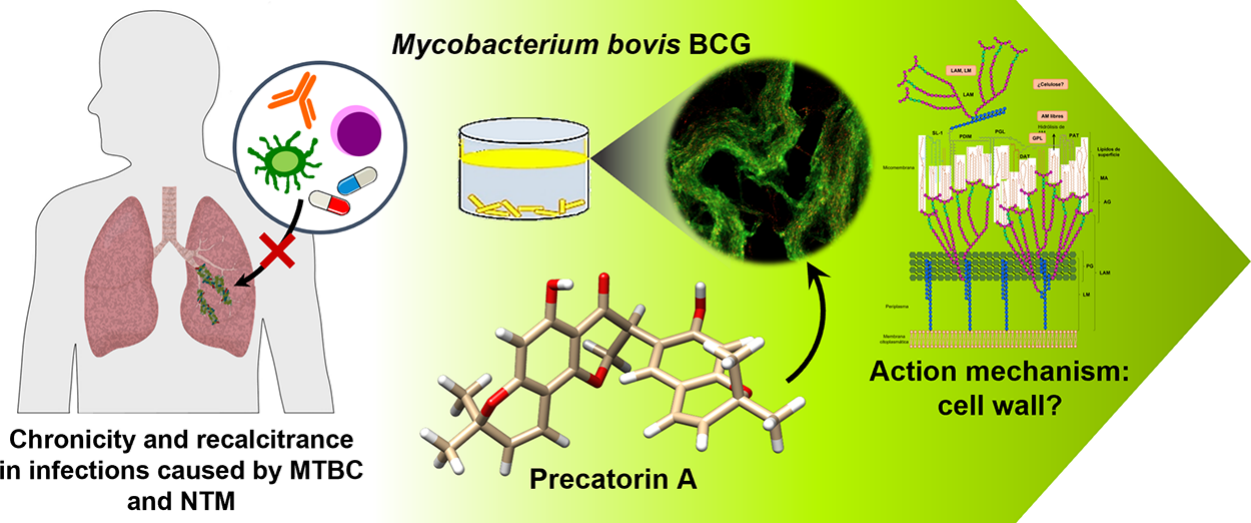

The aim of this study was to evaluate the potential antibiofilm activity of *Rhynchosia precatoria* (*R. precatoria*) compounds over *Mycobacterium bovis* BCG (*M. bovis* BCG) as a model for *Mycobacterium tuberculosis* (*Mtb*). We evaluated the antibiofilm activity as the ability to both inhibit biofilm formation and disrupt preformed biofilms (bactericidal) of *R. precatoria* compounds, which have been previously described as being antimycobacterials against *Mtb*. *M. bovis* BCG developed air–liquid interface biofilms with surface attachment ability and drug tolerance. Of the *R. precatoria* extracts and compounds that were tested, precatorin A (PreA) displayed the best biofilm inhibitory activity, as evaluated by biofilm biomass quantification, viable cell count, and confocal and atomic force microscopy procedures. Furthermore, its combination with isoniazid at subinhibitory concentrations inhibited *M. bovis* BCG biofilm formation. Nonetheless, neither PreA nor the extract showed bactericidal effects. PreA is the *R. precatoria* compound responsible for biofilm inhibitory activity against *M. bovis* BCG.

## Introduction

Tuberculosis (TB), which is primarily caused by airborne *Mtb* infection, continues to be one of the main causes of death by an infectious agent worldwide.^1^ According to the World Health Organization, drug-resistant TB incidence increased in 2021, with 450,000 new cases of rifampicin-resistant TB (RR-TB). Furthermore, zoonotic TB elicited by *M. bovis* may exacerbate the issue, especially in neglected communities of both low- and high-income countries.^2^ Nontuberculous mycobacteria represent a comparable threat due to their ability to act as opportunistic pathogens and to colonize pulmonary tissues, wherein they exhibit the ability to develop biofilms with an increased tolerance to antimicrobial treatment compared with planktonic bacteria.^3−5^ Similarly, *Mtb* air–liquid interface pellicles have been previously described as being present in human lung cavities,^6,7^ and biofilm-like microcolonies have been found in the pulmonary tissues of experimental models,^8,9^ thus indicating that this form of growth may be a significant contributor to drug resistance and is thereby an essential issue to address.

Biofilm constitution and architecture provide a safe location for the bacterial cells. The main constituents of the matrix are exopolysaccharides accompanied by lipids, proteins, and nucleic acids, which provide a suitable structure for water and nutrient channel formation and a variety of viscoelastic properties that allow them to attach to surfaces, modulate growth and migration, and escape from mechanical, chemical, and biological effects, such as immune responses (phagocytosis, opsonization, and complement activation) and protozoa predation.^10−12^ Biofilm-embedded cells are strategically distributed and classified into subpopulations with diverse genetic and metabolic features, which are harmoniously coordinated to sustain the community, thus making it more resilient to environmental changes (i.e., changes in temperature, nutrient concentration, humidity, and gas availability).^13,14^ This genetic diversity is recognized as being an essential element in the development of tolerance, which can eventually lead to resistance in both *in vivo* and *in vitro* settings.^15,16^ Therefore, biofilm inhibition and disruption are considered alternative strategies to resolve this underlying issue.

Only a few antibiofilm compounds have demonstrated activity against mycobacterial species, most of them by targeting molecules associated with cell wall development,^17−22^ which is the major barrier of the mycobacteria. A significant number of metabolic pathways involved in cell wall lipid synthesis are responsible for biofilm composition, which predominantly include free mycolic acids that provide a hydrophobic barrier that impedes antimicrobial penetration and restricts the cellular and molecular mechanisms of the immune response.^23,24^ Additionally, cellulose may play an architectural role in biofilm development, whereas lipomannan and lipoarabinomannan increase structure stability and immunomodulation, altogether contributing to persistence.^25−27^ Therefore, the isolation and characterization of biofilm inhibitors or disruptors that simultaneously target cell wall synthesis are of great interest.^41^

A few molecules isolated from various natural sources display antibiofilm activity against a range of microorganisms.^28,29^ Recently, we isolated a group of novel and known isoflavonoid compounds from *R. precatoria* roots that display antimycobacterial activity (inhibitory and bactericidal over planktonic cells) against *Mtb* and *Mycobacterium smegmatis*; these compounds were named precatorin A (PreA), precatorin B (PreB), cajanone (Caj), and lupinifolin (Lup) and classified as either isoflavanones or flavanones.^34^ Lupinifolin, a prenylated flavanone, has been previously described with antibiofilm activity against *Enterococcus* and *Streptococcus* spp., as well as a potentiator of other antimicrobial drugs by altering the cell wall integrity of bacteria.^30,31^ However, the antibiofilm activity of *R. precatoria* compounds has not been evaluated; hence, the aim of this study was to evaluate the potential antibiofilm activity of *R. precatoria* compounds over *M. bovis* BCG (which is a member of the *Mtb* complex and is known as MTBC) as a model for *Mtb*.

## Materials and Methods

### Bacterial Strains and Growth Conditions

*M. bovis* BCG Pasteur 1173P2 was kindly provided by Clara Espitia (Instituto de Ciencias Biomédicas, UNAM). Bacteria were cultured in 7H10 Middlebrook/10% OADC (v/v, BD Difco) agar plates at 37 °C until reaching the logarithmic phase (12–14 days). Afterward, cell suspensions were prepared via mechanical disruption and adjusted to the No. 1 McFarland standard by using turbidimetry (184–186 NTU). For fREMA and biofilm formation assays, cell suspensions were diluted to 12 × 10^6^ cells mL^–1^ with 7H9 Middlebrook/10% OADC broth (7H9M/10% OADC). Strain manipulation was made in BLS-2 facilities according to the manufacturer’s instructions.^32,33^

### Antimycobacterial Compounds

Stock solutions of the commercial drugs rifampicin (RIF), isoniazid (INH), and ethambutol (EMB) were prepared according to the manufacturer’s instructions (Sigma-Aldrich). Stock solutions of dichloromethane *R. precatoria* extract (RPE) and its purified compounds PreA (Table 1) and Caj^34^ were dissolved in DMSO (Sigma-Aldrich) at 5 or 10 mg mL^–1^ and stored at 4 °C. Cells were treated at inhibitory or subinhibitory concentrations, as described in each experiment.

**Table 1:**
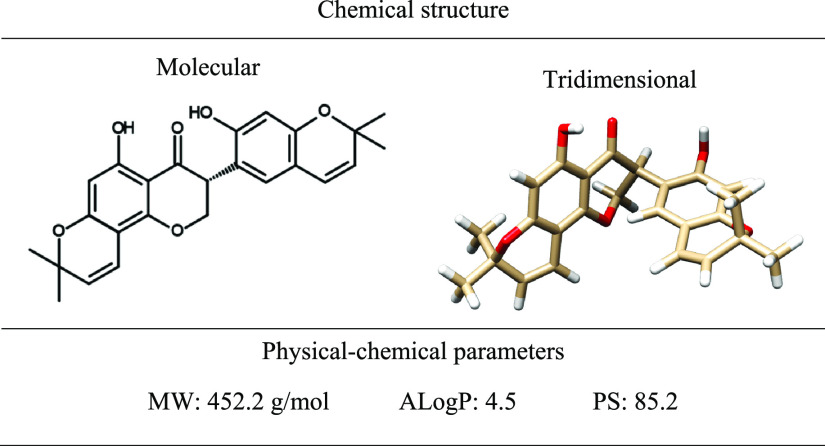
Physical–Chemical Features of the Precatorin A Compound^a^

a Chemical structures were created with USCF Chimera v.1.15.0. based on published data.^34^ Physical–chemical parameters were calculated with BIOVIA Draw 2017. MW: molecular weight; ALogP: partition coefficient; PS: polar surface.

### Antimycobacterial Activity Determination

**Figure 1:**
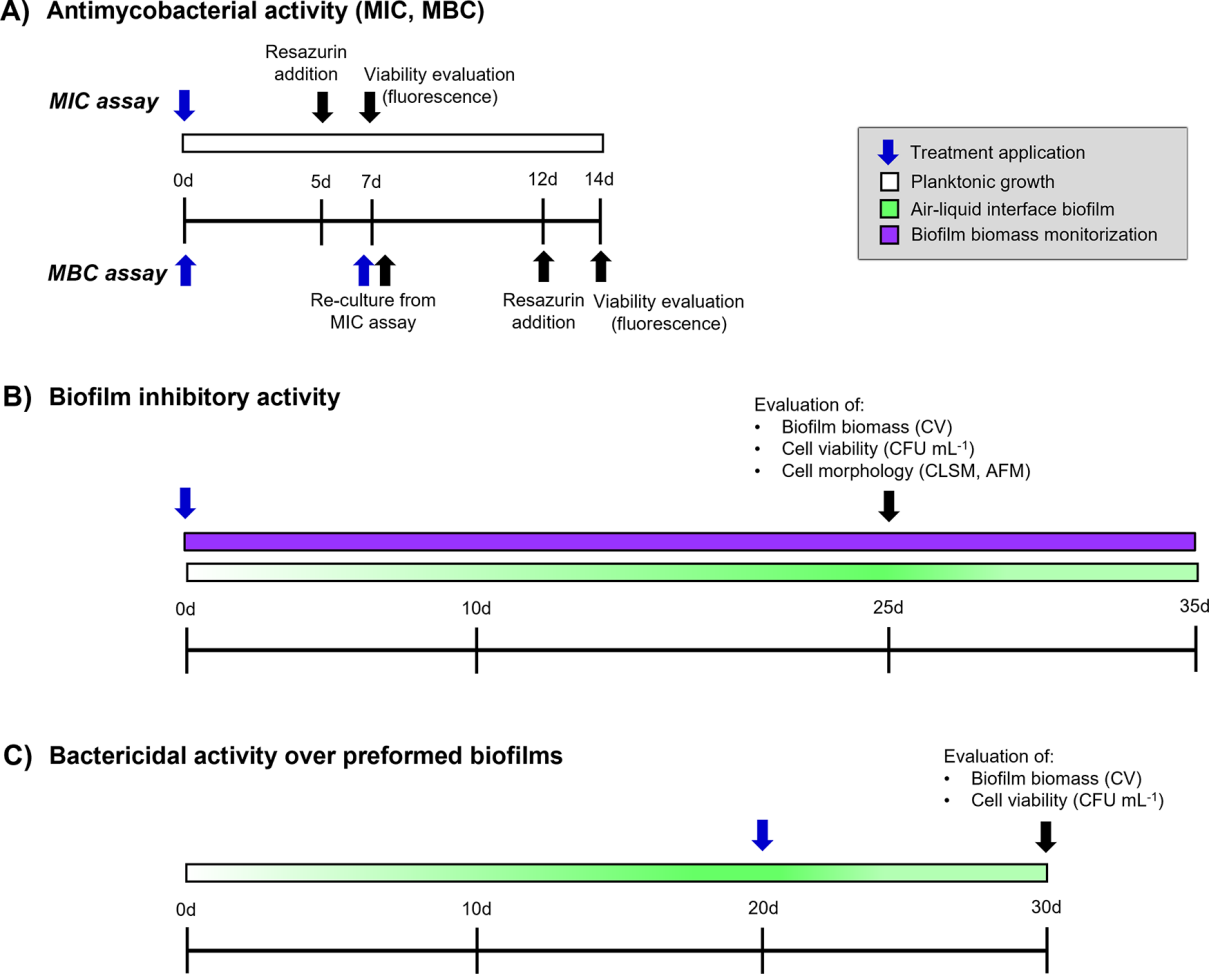
Experimental timeline for antibiofilm activity evaluation on *M. bovis* BCG. Antimycobacterial activity of commercial drugs and *R. precatoria* extract and its compounds was determined via the fREMA assay as shown in (A). Biofilm inhibitory and bactericidal activities of the antimycobacterials were evaluated by determining biofilm biomass and cell viability at the times indicated in (B) and (C); cell morphology was analyzed in biofilm inhibitory assays (C). For evaluation of biofilm inhibitory activity, bacteria were cultured at the MIC of the antimycobacterials, unless otherwise indicated. For bactericidal activity, commercial drugs (RIF, INH, and EMB) were used at MIC, MICx10, and MICx100; RPE, PreA, and Caj at MIC. MBC, minimal bactericidal concentration; fREMA, fluorometric resazurin microplate assay; CV, crystal violet staining; CFU mL^–1^, colony forming units per milliliter; CLSM, confocal laser scanning microscopy; AFM, atomic force microscopy; and d, days.

Minimum inhibitory and bactericidal concentrations (MIC and MBC, respectively) were determined via microdilution fREMA (fluorometric resazurin microplate assay) for mycobacteria.^35^ Briefly, bacteria (12 × 10^6^ cells mL^–1^) were cultured in 7H9M/10% OADC in the presence of serially diluted commercial drugs (RIF, INH, and EMB), RPE, PreA, or Caj in flat-bottom 96-well plates (Corning) at a final volume of 200 μL. Commercial drug concentrations ranged from 0.06 to 60 μg mL^–1^, and RPE and its compounds ranged from 0.5 to 250 μg mL^–1^. For maintenance of humidity, water was added to the outer wells, and plates were covered with plastic bags. Resazurin (0.01% w/v, Sigma–Aldrich) was added on the fifth day of culture; after 2 days, fluorescence readings were taken in a ThermoScientific Fluoroskan Ascent at λ_exc_/_em_ 485/538. MIC was defined as the minimal concentration of the antimycobacterial in which the well failed to show an increase in fluorescence in concordance with the nonbacteria control. For MBC determination, 5 μL of the MIC assay (no resazurin added) was transferred to new plates containing fresh 7H9M/10% OADC and serially diluted antimycobacterials, and the assay was performed under the same conditions. MBC was indicated as the minimal concentration of the antimycobacterial in which the well failed to show an increase in fluorescence in concordance with the nonbacterial control after reculturing the biomaterial in MIC assays. Figure 1 shows the experimental timeline of this study.

### Antibiofilm Activity Assays

Air–liquid interface biofilms were generated in 96-well plates under the growth conditions that were previously mentioned (12 × 10^6^ cells mL^–1^ in 7H9 Middlebrook/10% OADC broth, plastic sealed). The biofilm inhibitory activity of the commercial drugs RPE, PreA, and Caj was evaluated at MIC (Table 2) and monitored for 35 days; moreover, a detailed analysis of biofilm biomass and cell viability (both planktonic and biofilm) was assessed at Day 25. The bactericidal effect was evaluated over preformed air–liquid interface biofilms. Planktonic cells were discarded, and biofilms were treated either individually or combined at different concentrations, both inhibitory (MIC) and superinhibitory (MICx10 and MICx100) for the commercial drugs and inhibitory for RPE, PreA, and Caj. After 10 days of treatment, the biomass and cell viability of the biofilms were assessed by using the techniques mentioned below.

**Table 2:** Antimycobacterial Susceptibility Profiles of *M. bovis* BCG^a^

Antimycobacterial	*M. bovis* BCG Pasteur 1173P2	
	MIC μg mL^–1^ (μM)	MBC μg mL^–1^ (μM)
RIF	0.06 (0.07)	0.27 (0.29)
INH	0.20 (1.46)	0.20 (1.46)
EMB	3.13 (11.30)	2.35 (8.63)
RPE	31.25	62.5
PreA	31.25 (69.10)	31.25 (69.10)
Caj	31.25 (74.01)	31.25 (74.01)

a MIC and MIB were determined via the fREMA assay. RIF: rifampicin; INH: isoniazid; EMB: ethambutol; RPE: *R. precatoria* extract; PreA: precatorin A; Caj: cajanone.

### Biofilm Biomass Quantification

Biofilms obtained from the inhibitory and bactericidal assays were washed 3 times with triple-distilled water, stained with crystal violet (0.1% w/v, Sigma-Aldrich) for 15 min, and then washed three times. Dry crystals were dissolved in 33% acetic acid (v/v) and later quantified via spectrophotometry at 570 nm (Thermo Scientific MultiSkan Go). Determinations were made at specific time points or every 5 days (Figure 1).

### Viable Cell Count

The viability of both planktonic and biofilm cells was determined using the colony-forming unit count method (CFU mL^–1^). Samples were harvested and washed with 7H9 Middlebrook broth (3.3 rcf, 5 min, 4 °C). Bacterial suspensions were prepared via mechanical disaggregation for 10 min in 7H9 broth and filtered with 31G needles (BD). Bacterial dilutions were plated in 7H10 Middlebrook/10% OADC agar and cultured for up to 18 days.

### Confocal Laser Scanning Microscopy (CLSM)

Biofilm samples from the inhibitory assays were transferred and analyzed (either unstained or double-stained). Staining was performed with 5 μM CFDA-SE (Sigma-Aldrich) for 20 min at 37 °C in the dark. After a triple-distilled water wash, 2.5 μM propidium iodide (PI) was added for 10 min at 4 °C, washed three times, fixed with 10% formaldehyde (FA) (Sigma-Aldrich), and washed again. Sample examination was conducted with a Carl Zeiss Axio Observer Z1/7 microscope coupled with an LSM800 laser unit, using λ_exc_/_em_ 495/519 for CFDA-SE and λ_exc_/_em_ 305/617 for PI. Additionally, micrographs were taken with Plan Achromat 20×/0.8 M27 objective, and images were size scaled to 232 × 232 μm. Image visualization and analysis were performed with Zen Blue software v2.3.

### Atomic Force Microscopy (AFM)

Biofilm samples from the inhibitory assays were collected from the 96-well plates, placed in glass slides, washed, and fixed with 10% FA for 30 min. Sample examination was conducted in a Raman-AFM Alpha 300RA instrument (WiTec) in noncontact mode. Topographic and phase examinations were conducted in 50 × 50 μm areas with a silicon nitride cantilever of 42 N/m spring constant and a resonance frequency of 285 kH. Furthermore, WITec software was used for sample analysis and processing. The results are shown as the root-mean-square height (SQ) in nm.

### Statistical Analysis

Data from three independent experiments that were performed with at least three technical replicates are presented as the mean and standard deviation. Comparisons between the groups were conducted via one-way ANOVA with a post hoc Tukey test (*p* ≤ 0.05).

## Results and Discussion

### Antimycobacterial Drug Susceptibility Profile of *Mycobacterium bovis* BCG

*M. bovis* BCG was used as a model for the evaluation of the possible antibiofilm effects of RIF, INH, EMB, RPE, PreA, and Caj on *Mtb,* for which the MIC and MBC were determined on planktonic cells (Figure 1). *M. bovis* BCG demonstrated a susceptibility profile similar to that of *Mtb* H37Rv to commercial drugs, RPE, PreA, and Caj (Table 2, 32). RIF was the most active drug over planktonic growth while EMB the least effective, with an active concentration ranging from 0.06 to 3.13 μg mL^–1^, a comparable value to that of *Mtb* H37Rv previously observed.^34^ Additionally, RPE, PreA, and Caj also displayed similar activities to that of *Mtb* (31.25 to 62.5 μg mL^–1^), however not as active as the commercial drugs (Table 2,^32^).

*M. bovis* BCG is one of the most commonly used models in the search for antituberculous drugs. This is due to being a member of the MTBC, thereby displaying a similar physical and metabolic behavior to *Mtb*, although there are some differences in the mycolic acid layer and protein composition.^36−38^ The obtained drug susceptibility profile reflects such similarities and the applicability of BCG as a *Mtb* model,^34^ even more so when considering the spectra of bacterial targets (such as transcription and cell wall synthesis).^39,40^ More importantly, BCG was shown to be susceptible to RPE, PreA, and Caj, which supports the choice of this strain to determine antibiofilm activities in this study.^36^ Furthermore, experimentation with the attenuated strain *M. bovis* BCG requires a lower biosafety level (BSL-2) which facilitates the execution of hazardous technical procedures used, such as biofilm disaggregation leading to potential aerosol formation and culture microscopic analysis.

### General Features of Mycobacterial Air–Liquid Interface Biofilms

**Figure 2:**
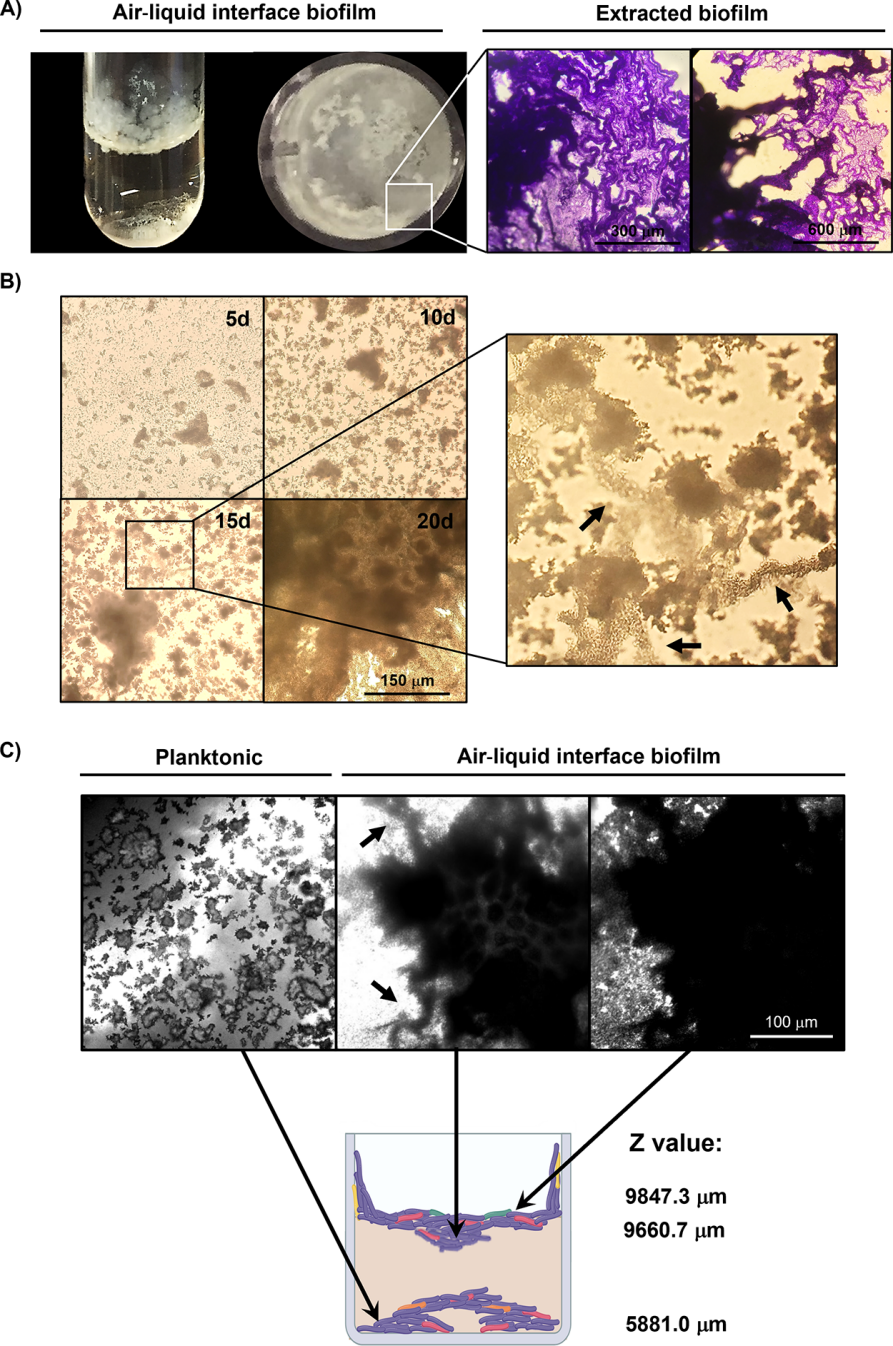
*M. bovis* BCG air–liquid interface biofilm formation. BCG cultured in Middlebrook 7H9 broth supplemented with 10% OADC for 25 days resulted in adherent and highly heterogeneous pellicle formation. Biofilm extraction and staining allowed for the visualization of heavy cording and thick biofilm areas (optic microscopy, 10×) (A). Planktonic cell aggregation facilitated microcolony formation and subsequent air–liquid interface biofilm formation (optic microscopy, 20×) (B). Both planktonic and biofilm strata can be identified by using CLSM (20×) (C).

*M. bovis* BCG was able to develop air–liquid interface biofilm cultures under static conditions, which were highly adherent to plastic surfaces and glass (Figure 2A). According to the observations, precipitated and aggregated planktonic mycobacteria produced irregular microcolonies that were later integrated by association with cording structures, including the development of pellicles (Figure 2A,B). Colonies later increased their size, thickness, cell density, and complexity to form air–liquid interface biofilms, whereas some planktonic cell populations remained at the well bottom (Figure 2B,C). Bacterial cultures were also visualized by using confocal microscopy, which confirmed the existence of the different strata of which the upper (biofilm) was highly heterogeneous in morphology and thickness (Figure 2C). Moreover, heavy cording was also detected in air–liquid interface biofilms (Figure 2A,C). The layouts of both planktonic and air–liquid interface growth that were exhibited in this model facilitated their segregation for biofilm biomass quantification, individual cell quantification, and morphologic analysis (confocal and atomic force microscopies).

The utilized media provided a rich source of C and N for mycobacterial development in an environment similar to that found in lung cavitation, wherein pellicle structures have been previously detected.^6,7^*In vitro* pellicle formation can be used to study biofilm spreading toward and across the surface, which is an essential feature in tissue colonization. Furthermore, the energetic metabolism behavior, macromolecule distribution, and drug persistence can be assessed under appropriate conditions.

These properties are valuable for modeling in a static microenvironment such as the lung; however, it would be necessary to complement this matrix by adding other components, such as epithelial and immune cells.^9^

### Biofilm Inhibitory Activity of *R. precatoria* Extract and Its Compounds

**Figure 3:**
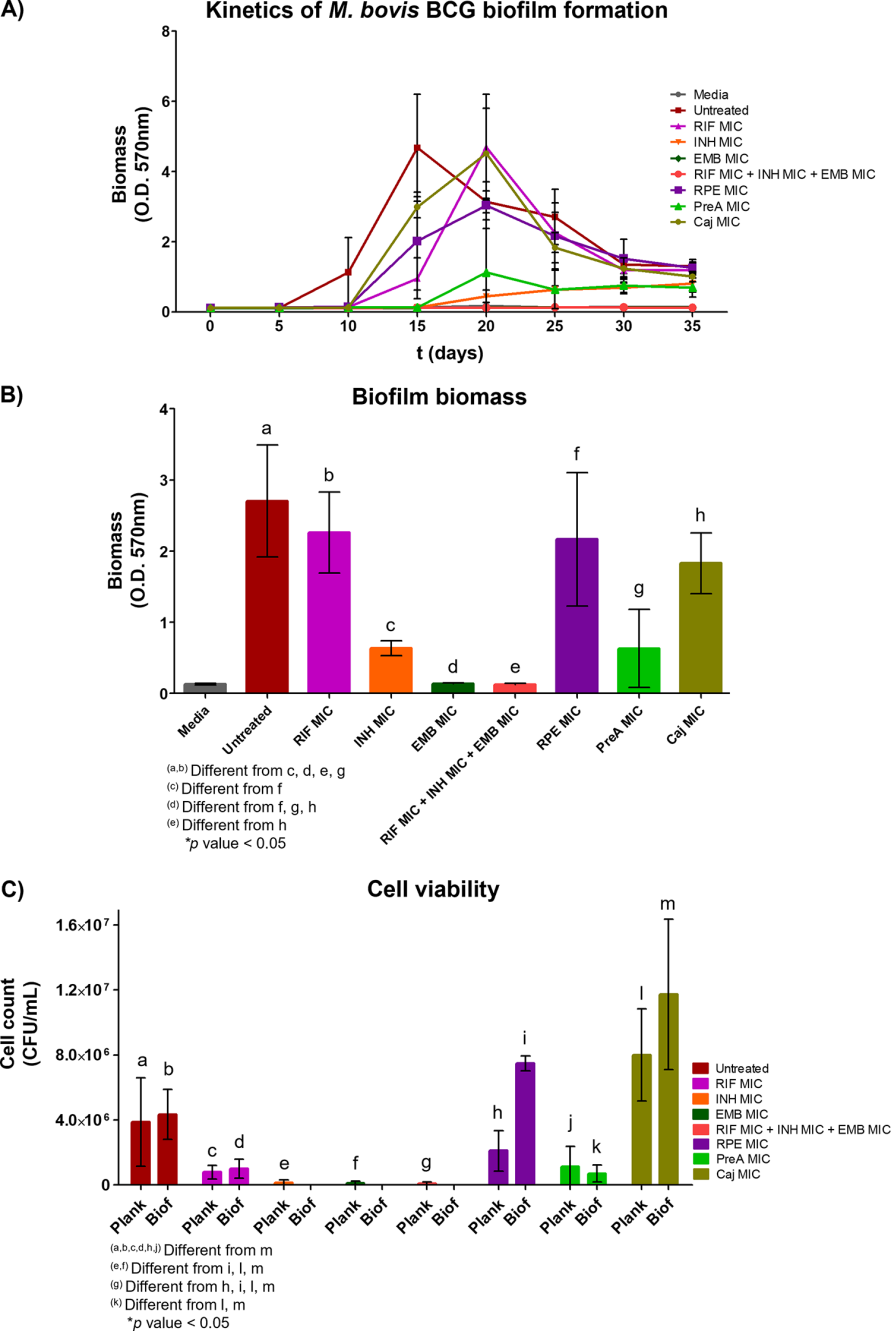
Biofilm inhibitory activity of antimycobacterials against *M. bovis* BCG. Bacilli were cultured in the presence of *R. precatoria* extract (RPE), PreA, and Caj at 31.25 μg mL^–1^; rifampicin (RIF) at 0.0625 μg mL^–1^, isoniazid (INH) at 0.20 μg mL^–1^, and ethambutol (EMB) at 3.13 μg mL^–1^. Kinetics of biofilm formation was evaluated with crystal violet staining for up to 35 days (A). For inhibitory activity evaluation, biofilm biomass and cell viability were determined at Day 25 (B, C). Representative results of three independent experiments; media and error bars (SD) are shown. Differences between treatments were assessed by using one-way analysis of variance (ANOVA) with Tukey’s post hoc multiple comparison test (*p* < 0.05).

To evaluate the potential inhibitory activity of RPE and its compounds against *M. bovis* BCG and biofilms, bacteria were cultured at MICs (Table 1) under the above-mentioned conditions. Kinetic biofilm formation was monitored by using crystal violet staining (Figures 1 and 3).

Untreated *M. bovis* BCG developed an air–liquid interface biofilm at Day 10, reaching its maximum level at Day 15 with an OD_570 nm_ of 4.7 ± 1.5. RPE and Caj delayed biofilm formation to Day 20, with maximum levels of OD_570 nm_ 3.0 ± 0.7 and OD_570 nm_ 4.5 ± 1.3, respectively. Interestingly, PreA decreased biomass production to the order of OD_570 nm_ 1.1 ± 1.5, in addition to delaying biofilm formation at Day 20 (Figure 3A). RIF was not able to inhibit air–liquid interface biofilm formation, although INH and EMB did inhibit this effect. Only these three drugs combined at MIC were able to inhibit biofilm formation with an OD_570 nm_ of 0.1 ± 0.0 (Figure 3A).

A detailed analysis of biomass and cell viability at Day 25 showed that PreA inhibited biofilm formation (biomass) to an extent of 76.6% compared to the untreated control (*p* < 0.05) and was more active than RPE and Caj (19.7 and 32.2%, respectively) (Figure 3B). For the drugs (MIC), INH, EMB and the three drugs combined (RIF, INH, and EMB) significantly reduced biofilm formation (*p* < 0.05) with 76.5, 95.1, and 95.4%. respectively; however, RIF showed a 16.2% reduction (nonsignificant) (Figure 3B).

In regard to viable cell count, PreA caused a 76.2% (7 × 10^5^ ± 5.2 × 10^5^ CFU mL^–1^) inhibition of biofilm growth against the untreated control (4.3 × 10^6^ ± 1.5 × 10^6^ CFU mL^–1^) (*p* < 0.05, no statistical significance) (Figure 3C); however, it is important to note its consistency with the biofilm biomass inhibition (76.6%). Additionally, PreA proved to be the most active RPE compound, due to the fact that it inhibited biofilm cell viability in contrast with Caj (*p* < 0.05). The ability of INH, EMB, and RIF + INH + EMB to completely inhibit biofilm cells confirmed their role as the best biofilm inhibitors (Figure 3C).

The experiments demonstrated the predominance of PreA as the RPE biofilm inhibitor compound on *M. bovis* BCG, with the possibility of exhibiting a similar effect on *Mtb* H37Rv because of its similar antimycobacterial susceptibility profile and physiology. The observed inhibitory effect was transient due to the fact that *M. bovis* BCG was able to develop biofilms later in time in the presence of PreA, as observed with other treatments such as RIF, RPE, and Caj (Figure 3A,B). This effect was likely caused by the remaining drug-tolerant cell populations that either proliferated or developed the ability to act as biofilm high producers, potentially as a protection mechanism to strengthen the microbial community.^41^

For the commercial drugs, it is important to highlight the fact that BCG developed biofilms in the presence of RIF, which is the most active drug against *Mtb*, in agreement with reports in which mycobacteria developed a drug-tolerant biofilm upon their exposure to this drug through mechanisms related to *rpoB* upregulation and cell division.^39,42^ In contrast, INH was able to deplete biofilm development, thus confirming the importance that mycolic acids possess in the extracellular matrix composition;^23,43,44^ however, the detected planktonic cell proliferation also indicated the emergence of drug-tolerant populations that may eventually become resistant. Finally, EMB was shown to be crucial in biofilm inhibition, which can be explained by its role in interrupting arabinosyltransferase activity, thus affecting the cell wall stability and lipoarabinomannan arabinosylation located in the mycobacterial capsule.^45,46^ Nevertheless, the three drugs combined showed the best biofilm inhibitory and bactericidal effects (Figures 3 and 6).

**Figure 4:**
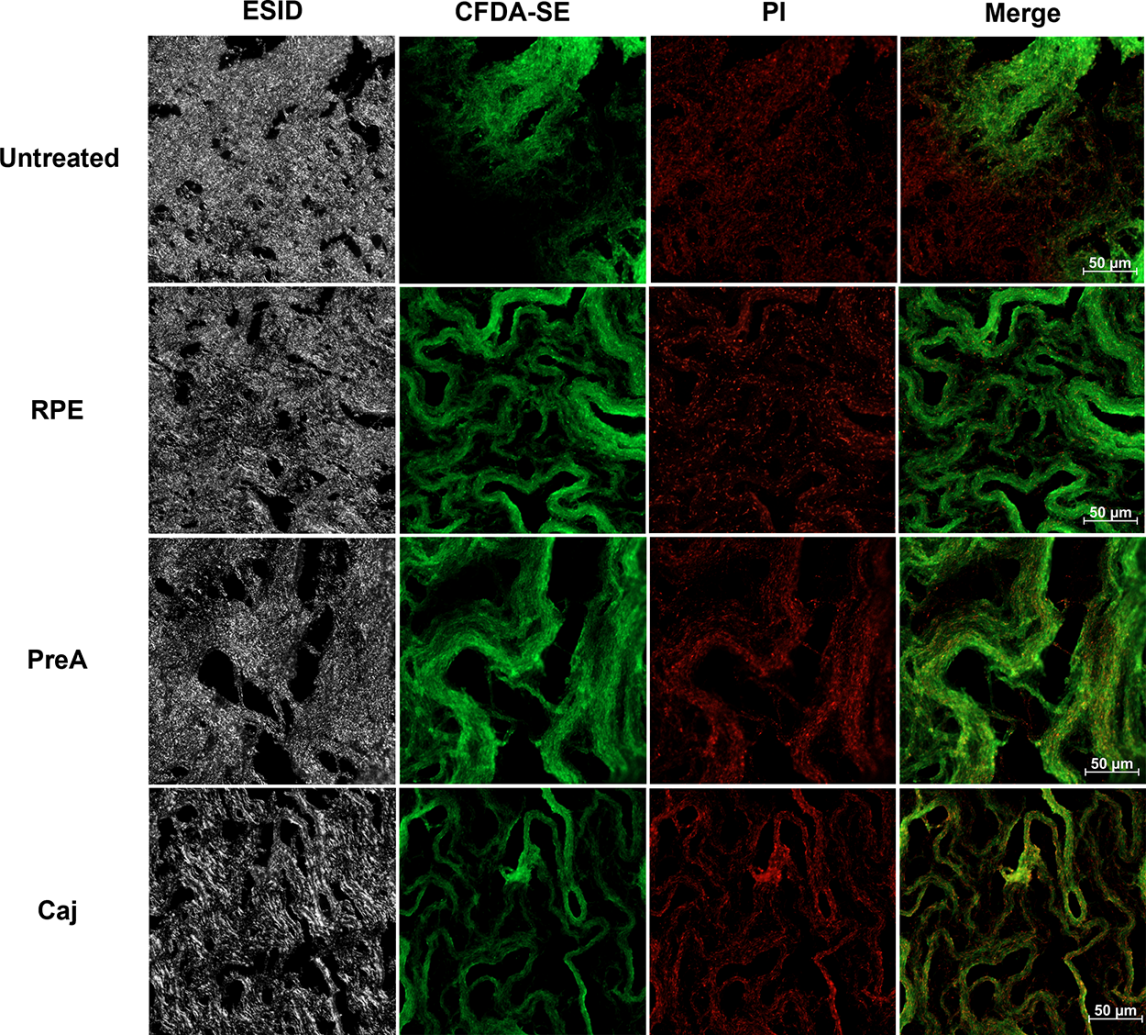
Distribution of live and dead cells in air–liquid interface biofilms. Twenty-one-day-old *M. bovis* BCG biofilms generated in the presence of RPE, PreA, and Caj were double-stained with 5 μM CFDA-SE and 2.5 μM PI and analyzed by using CLSM. Micrographs were taken with a 20×/0.8 M27 objective, and images were size scaled to 232 × 232 μm. Representative results of three independent experiments are shown. ESID, electronically switchable illumination and detection; CFDA-SE, carboxyfluorescein succinimidyl ester; and PI, propidium iodide.

Biofilm samples were collected at 20–25 days of culture and analyzed via CLSM and AFM. For the CLSM analysis, CFDA-SE/PI double-stained samples allowed us to analyze the cell distribution in biofilms. The RPE, PreA, and Caj treatments induced the formation of a more heterogeneous biofilm in which coding structures could be easily detected in comparison with the untreated control. Moreover, it is possible that this heavy cording was associated with the increase in matrix production (i.e., exopolysaccharides, lipids, and nucleic acids), given the considerable amount of unstained material that was detected, as shown by the ESID images (Figure 4). Additionally, classic biofilm ultrastructures could be visualized as nutrient and water channels, along with cords, potentially unifying microcolonies in a way to expand the biofilm community. Furthermore, live and dead cells seemed to be homogeneously distributed in treated biofilms compared with those in untreated biofilms (Figure 4).

**Figure 5:**
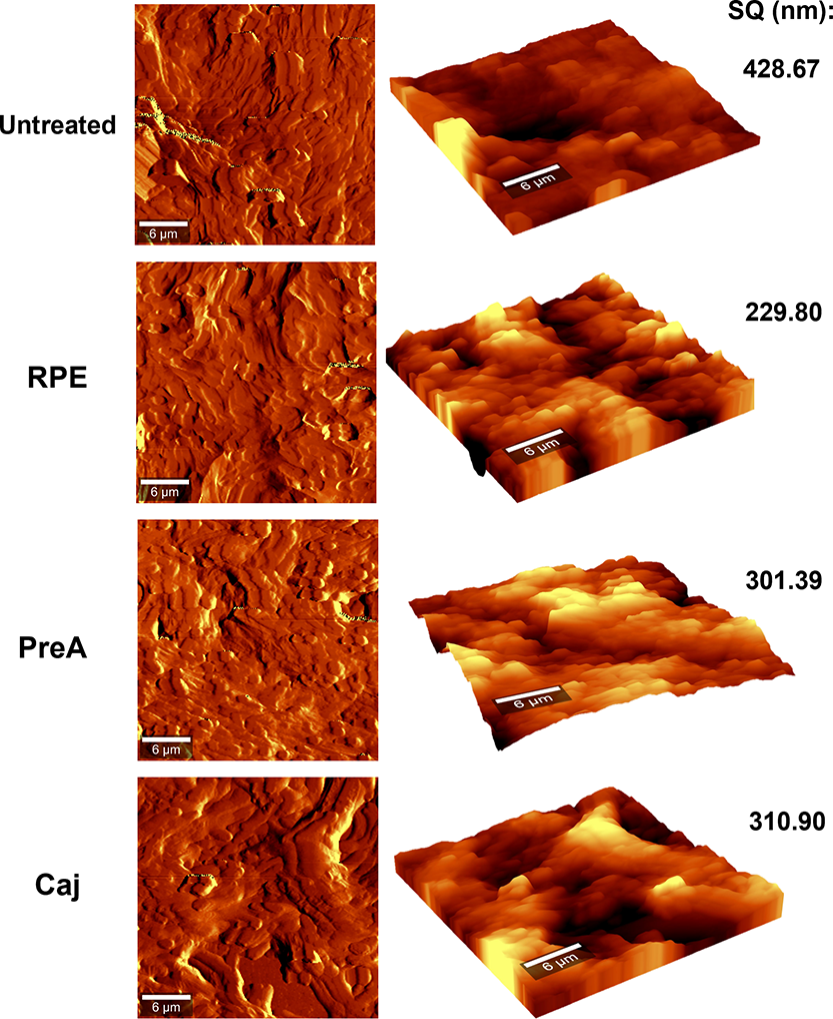
Roughness (SQ) variation of *M. bovis* BCG air–liquid interface biofilms developed in the presence of RPE, PreA, and Caj. Twenty-five-day-old biofilms were collected, washed, and fixed for AFM analysis in noncontact mode. Root mean square height (SQ) values are shown in nm. AFM, atomic force microscopy.

Additionally, BCG biofilm analysis via AFM exhibited a decrease in the root-mean-square height (SQ) upon treatment with RPE, PreA, and Caj (Figure 5), thus demonstrating a loss of roughness. RPE decreased the SQ to 229.80 nm, PreA to 301.39 nm, and Caj to 310.90 nm, in comparison with 428.67 nm observed in the untreated biofilm. Whether this loss of roughness was due to alterations in macromolecule nature or their concentration in the matrix (or if it was caused by a reduction in viable cell number) is yet to be investigated. It will also be important to analyze the cell wall topography to evaluate a possible effect at the nano level.

### Bactericidal Activity of *R. precatoria* Extract and Its Compounds on Preformed Biofilms

**Figure 6:**
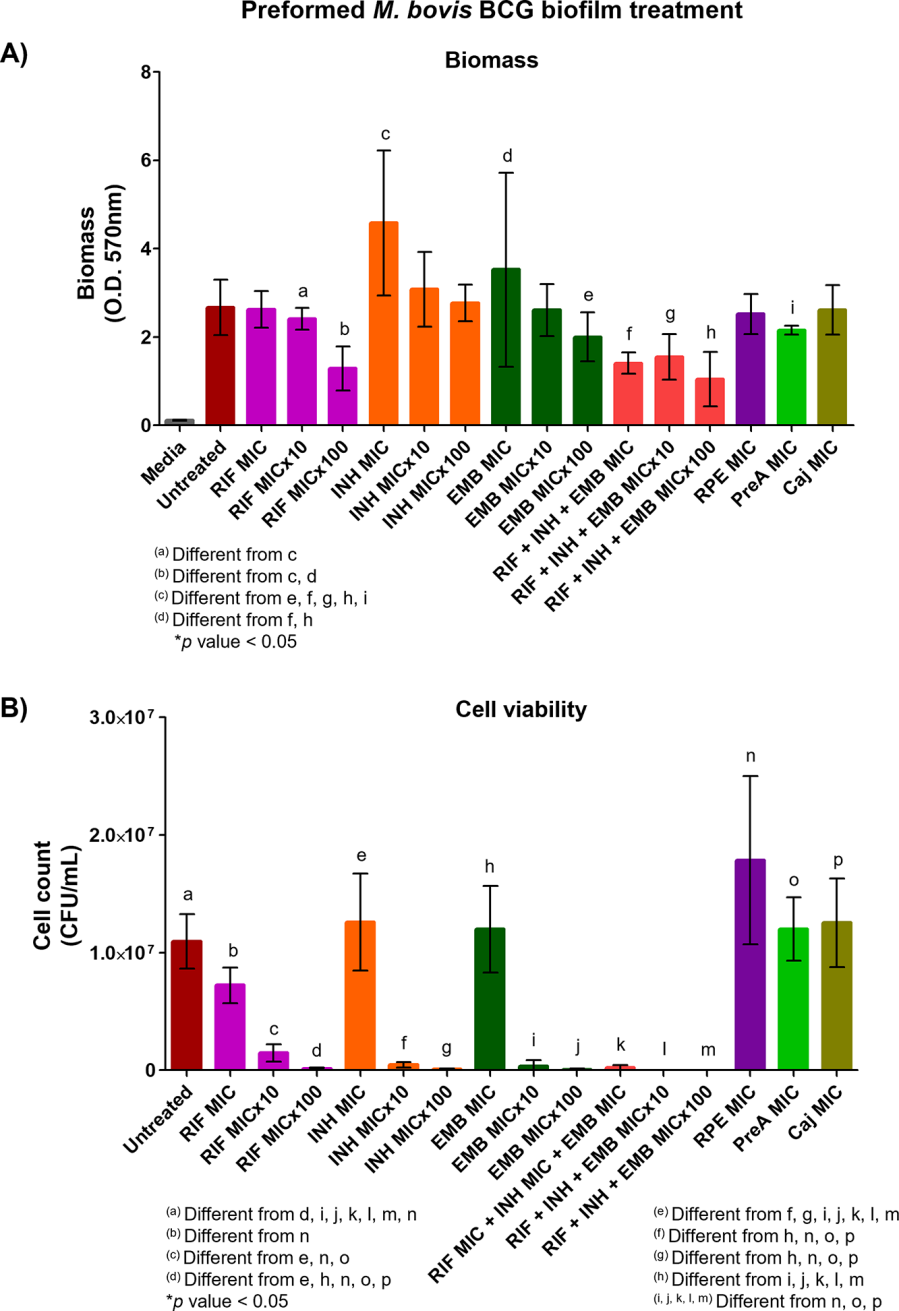
Bactericidal activity over the *M. bovis* BCG air–liquid interface preformed biofilms. Twenty-day-old preformed biofilms were treated with RPE, PreA, or Caj at MIC or RIF, INH, and EMB at inhibitory (MIC) and superinhibitory concentrations (MIC×10 and MIC×100), either alone or in combination. Analysis of biomass (A) and cell viability (B) was performed after 10 days of treatment. Representative results of three independent experiments; media and error bars (SD) are shown. Differences between treatments were assessed by using one-way analysis of variance (ANOVA) with Tukey’s post hoc multiple comparison test (*p* < 0.05).

To evaluate the possible bactericidal activity of RPE and its compounds over preformed air–liquid interface biofilms, planktonic cell cultures were placed for the development of homogeneous biofilms for 20 days (Figure 1). Biomass quantification demonstrated that neither RPE, PreA, nor Caj decreased the biomass of preformed biofilms as the commercial drugs did, either alone or in combination (Figure 6A). Even more, INH and EMB induced biofilm hyperproduction at MIC (in comparison with RIF) at superinhibitory concentrations (*p* < 0.05). INH (MIC) was also responsible for the increase in biofilm biomass compared with EMB (MIC 100×) and RIF + INH + EMB at both inhibitory and superinhibitory concentrations (*p* < 0.05) (Figure 6A). Nonetheless, viable cell count experiments demonstrated that RPE increased cell proliferation in biofilms compared with the untreated control, RIF (both inhibitory and superinhibitory concentrations), INH, and EMB (superinhibitory concentration), as well as a combination of the three drugs (inhibitory and superinhibitory concentrations) (*p* < 0.05). Moreover, PreA and Caj were shown to increase the viable cell count (Figure 6B). In contrast, the antimycobacterial drugs were highly effective at superinhibitory concentrations against biofilms (*p* < 0.05) (Figure 6B). These results indicate the need for at least three first-line treatment drugs for the eradication of MTBC members, even more so for a sustained treatment that enables superinhibitory drug concentrations that allow for the penetration of possible *in vivo* biofilms.

On this basis, the null bactericidal activity of PreA over preformed biofilms may have been derived from its inability to surpass the biofilm matrix. This observation is in agreement with the ability of biofilms to provide protection against antimicrobials, in contrast to planktonic cells, which reflect the biofilm matrix contribution to *in vitro* first-line drug tolerance.^4,47^ Even more, molecular changes at the unicellular level should not be neglected, given that the mycobacterial cell wall is a dynamic structure that varies its composition and fluidity as a consequence of metabolic changes.^48−50^

### PreA Inhibitory Additive Effect on First-Line Antimycobacterial Drugs

**Figure 7:**
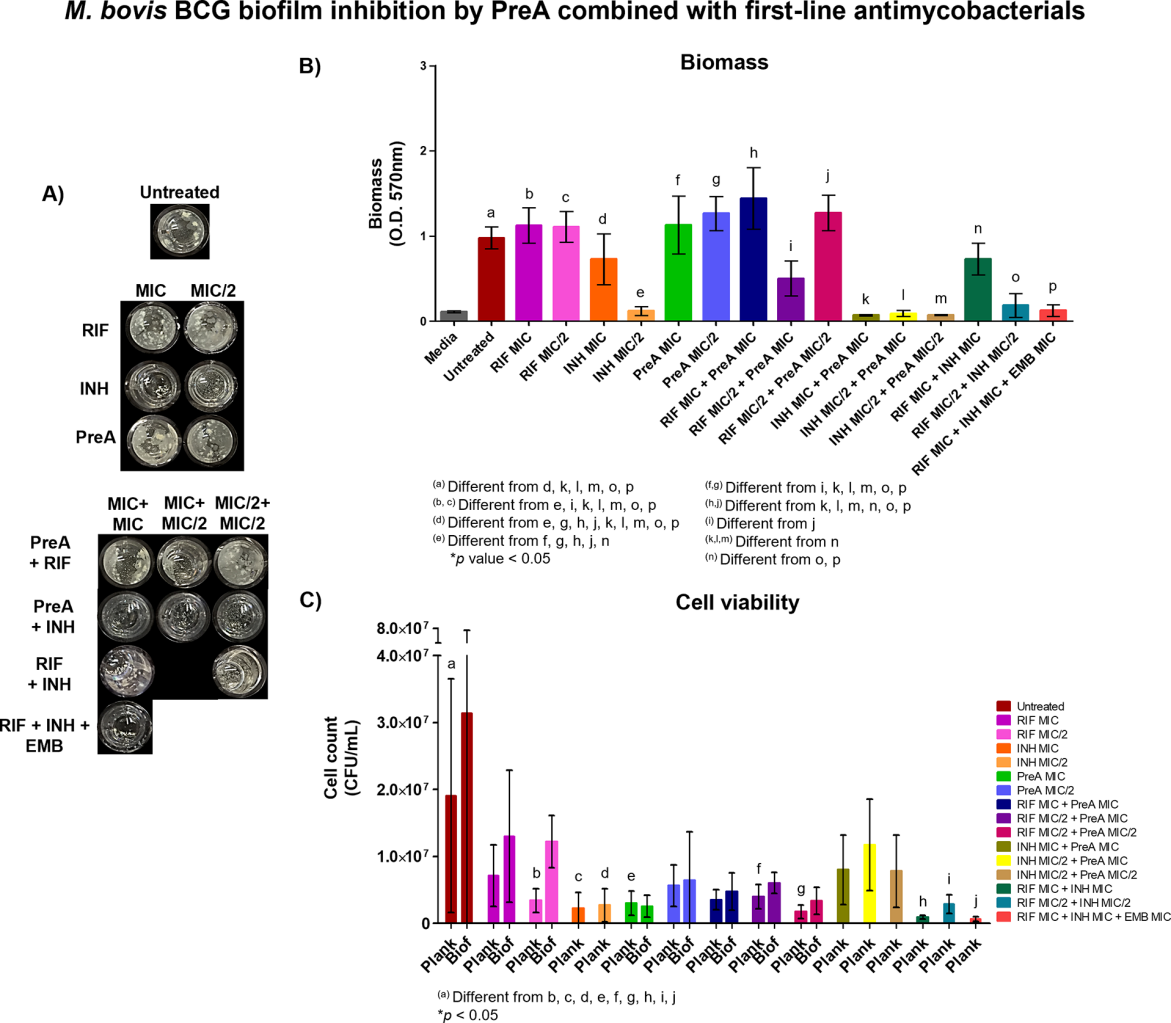
Biofilm inhibitory activity of PreA in combination with antimycobacterial drugs. *M. bovis* BCG was cultured in the presence of PreA and RIF or INH at inhibitory (MIC) or subinhibitory (MIC/2) concentrations for 20 d. Morphological observations were made (A), as well as biofilm biomass quantification (B) and cell viability (C). The combination of RIF, INH, and EMB at the MIC was used as the maximum inhibition control. Representative results of three independent experiments; media and error bars (SD) are shown. Differences between treatments were analyzed by using one-way analysis of variance (ANOVA) with Tukey’s post hoc multiple comparison test (*p* < 0.05).

To evaluate the potential of PreA as a biofilm inhibitory molecule along with other first-line antimycobacterials, experiments were performed by using the compound in combination with RIF or INH. As observed, the combination of PreA and INH at inhibitory and subinhibitory concentrations inhibited air–liquid interface biofilm development (*p* < 0.05) (Figure 7A,B). This effect was possibly due to the influence of INH because a similar result was observed in the RIF+INH controls (Figure 7A). However, these treatments did not have an effect on planktonic cell viability compared to the untreated control, which indicated that this condition inhibited only biofilm formation and not planktonic cell proliferation (Figure 7C).

The combination PreA (MIC) + RIF (MIC/2) significantly decreased biofilm formation in contrast to RIF (MIC) and RIF (MIC/2), possibly counteracting its effect on drug tolerance development.^39^ Of the three commercial drugs, INH was the only type that inhibited air–liquid interface biofilm formation. As in previous experiments, a combination of the three drugs RIF + INH + EMB at MIC completely hampered biofilm development (*p* < 0.05).

It is important to highlight the morphological differences between the INH (MIC) control (in which heavy flocculates were detected) and the three PreA + INH treatments (in which the bacterial aggregate size decreased) (Figure 7A). This PreA effect may promote air–liquid interface biofilm formation, perhaps as a bacterial defense mechanism to counteract its antimicrobial activity, as observed with RIF, which leads to the necessity to identify its targets and thereby learn about the extent of bacterial physiological impairment and potential medical use.

Due to the fact that PreA is a recently described compound, there have been no studies that provide information about its mechanism of action, although there are a number of flavonoids with reported antimycobacterial activity. PreA is an isoflavanone, which is a subgroup of the phenolic compounds that have shown inhibitory activity against *Mycobacterium* spp. in a variety of mechanisms.^51−53^ PreA is structurally related to the other accompanying compounds in the RPE (the isoflavanones precatorin B, precatorin C, and Caj) and the prenylated flavanone Lupinifolin (Lup).^34^ Unlike PreA, Caj and Lup have been identified in several plant genera and have shown antimicrobial activity against several bacterial species.^30,54,55^ Caj is the most abundant isoflavanone in *Cajanus cajan*, which is a leguminous plant widely consumed for its medicinal and nutritional properties, given its high content of a variety of flavonoids.^54,56,57^ Moreover, Caj is a prenylated isoflavanone that exhibits antifungal activity against *Fusarium oxysporum*,^58^ which is a *C. cajan* pathogen; however, no antibiofilm activity has been reported to date. It is believed that plants activate their biosynthetic metabolism to increase isoflavonoid production as a response to pathogenic microorganisms, such as fungi,^59,60^ that harbor a complex and thick cell wall, which is a feature common to mycobacteria.^61,62^ Moreover, due to their hydrophobic nature, isoflavonoids accumulate in plant cell walls as a response to bacterial infections and environmental stress^63,64^ as a means to inhibit unwanted microbial colonization, reinforce the cell wall structure, and favor symbiotic interactions.^59,65^ These hypothetical mechanisms may be explained by Lup activities. Studies have suggested that Lup disrupts the cell walls and membranes of Gram-positive microorganisms, thus increasing permeability and decreasing membrane potential, which eventually leads to cell leakage.^30,55,66^ Moreover, it has demonstrated antibiofilm activity and improved methicillin-resistant *S. aureus* (MRSA) sensitivity to ampicillin and cloxacillin,^30,31,67^ thus possibly having a synergistic effect by targeting a variety of cell mechanisms other than the cell wall and membrane, which is a common feature among flavonoids.^52,68^

It will be necessary to investigate whether PreA accumulates in the mycobacterial cell wall and the cytosol, as hypothesized for Caj and Lup, given that flavonoids are able to target a wide variety of molecules, thereby having an impact at multiple levels.^52^ Additional experiments will enable the identification of its target and mechanisms of action.

## Conclusions

PreA was identified as a biofilm inhibitor of *M. bovis* BCG, which is a member of the *Mtb* complex and one of the most commonly used models for potential antimycobacterials. The development of air–liquid interface biofilms was possible by using a simple static model, which contributed to the study of biofilm behavior in response to diverse antimicrobials including commercial drugs. Due to the identification of formation stages and strata, this model can be used for metabolic and genetic characterization of bacterial subpopulations (including tolerant and persistent) and micro- and macromolecule composition analysis.

It is crucial to investigate the mechanism of action of PreA, including whether it targets the mycobacterial cell wall (both its main protection barrier and its “Achilles heel”) through physical interactions or metabolic pathways related to their synthesis and assembly or their relationship with other bacterial mediators. Likewise, it is necessary to evaluate the response mechanisms to its treatment, including mainly those mechanisms related to counteracting its antimicrobial effects.

PreA is a flavonoid whose activity against *M. bovis* BCG could be improved through chemical modifications. Likewise, it is necessary to continue research for antimycobacterial flavonoids to fight resistant tuberculosis.

## Notes

All authors (E.I.N.-P., C.I.E., C.V., E.A., E.S.-C., A.B.-E., J.A.E.-M., O.V., H.A.-G., and A.G.-E.) have disclosed potential conflicts of interest.

## Abbreviations Used

BCG: Bacille Calmette-Guérin
Caj: cajanone
EMB: ethambutol
fREMA: fluorometric resazurin microplate assay
INH: isoniazid
Lup: lupinifolin
*Mtb*: *Mycobacterium tuberculosis*
MTBC: *Mycobacterium tuberculosis* complex
PreA: precatorin A
PreB: precatorin B
RIF: rifampicin
RPE: *Rynchosya precatoria* extract
RR-TB: rifampicin-resistant tuberculosis
TB: tuberculosis
